# GPT2 Is Induced by Hypoxia-Inducible Factor (HIF)-2 and Promotes Glioblastoma Growth

**DOI:** 10.3390/cells11162597

**Published:** 2022-08-20

**Authors:** Bo Zhang, Yan Chen, Lei Bao, Weibo Luo

**Affiliations:** 1 Department of Pathology, UT Southwestern Medical Center, Dallas, TX 75390, USA; 2 Department of Pharmacology, UT Southwestern Medical Center, Dallas, TX 75390, USA

**Keywords:** hypoxia, hypoxia-inducible factor, GPT2, glioblastoma, tumorigenesis

## Abstract

Hypoxia-inducible factor (HIF) directly activates the transcription of metabolic enzymes in response to hypoxia to reprogram cellular metabolism required for tumor cell proliferation. Through analyzing glutamate-linked aminotransferases, we here identified glutamate pyruvate transaminase 2 (*GPT2*) as a direct HIF-2 target gene in human glioblastoma (GBM). Hypoxia upregulated GPT2 mRNA and protein levels in GBM cells, which required HIF-2 but not HIF-1. HIF-2 directly bound to the hypoxia response element of the human *GPT2* gene, leading to its transcription in hypoxic GBM cells. GPT2 located at the nucleus and mitochondria and reduced α-ketoglutarate levels in GBM cells. Genetic or pharmacological inhibition of GPT2 decreased GBM cell growth and migration under normoxia and hypoxia. Knockout of GPT2 inhibited GBM tumor growth in mice. Collectively, these findings uncover a hypoxia-inducible aminotransferase GPT2 required for GBM progression.

## 1. Introduction

Glioblastoma (GBM) is a metabolic disease [1]. Glutamate regulates synapse plasticity, neurotoxicity, and edema in GBM patients and is a critical energy source that contributes to GBM cell survival and growth [2]. A group of aminotransferases including glutamate pyruvate transaminase (GPT, also known as alanine aminotransferase), glutamate oxaloacetate transaminase (GOT), branched-chain amino acid transaminase (BCAT), ornithine aminotransferase (OAT), and phosphoserine aminotransferase 1 (PSAT1) link glutamate with the tricarboxylic acid (TCA) cycle and amino acid metabolism, which are required for synthesis of macromolecular building blocks to support cancer cell proliferation [3,4,5]. Among these aminotransferases, GPT is known to localize to mitochondria and bridges glutamate metabolism with glycolysis, another essential metabolic pathway in GBM, by catalyzing a reversible transfer of an amino group from glutamate to pyruvate with production of alanine and α-ketoglutarate [6]. The GPT family consists of two isoforms, GPT1 and GPT2, and these two human isoforms share about 69% amino acid sequence identity [7]. GPT2 is a primary isoform expressed in brain. Loss of function mutations have been identified in the human *GPT2* gene and are associated with developmental encephalopathy, intellectual disability, and neurodegenerative disorders in humans [8,9]. A previous study showed that activating transcription factor 4 (ATF4) induces GPT2 expression in hepatic cells upon treatment of histidinol or tunicamycin [10]. GPT2 is also induced in prostate cancer cells treated with O-GlcNAc transferase inhibitor or androgen [11,12]. However, the regulatory mechanism of GPT2 expression in GBM and its role in GBM tumorigenesis remain unknown.

Hypoxia is frequently detected in the microenvironment of GBM and mediates GBM progression [13]. The adaptive responses to tumor hypoxia are primarily mediated by the transcription factors hypoxia-inducible factors (HIFs) [14]. Three HIF family members, HIF-1, HIF-2, and HIF-3, have been identified in mammals, each of which comprises an oxygen-regulated α subunit (HIF-1α, HIF-2α, or HIF-3α) and a constitutively expressed β subunit (HIF-1β or ARNT2) [15,16,17]. HIF-α is prolyl hydroxylated by a family of prolyl hydroxylase domain proteins (PHDs) in the presence of the substrate O_2_ and the cofactors α-ketoglutarate, iron, and ascorbate [18,19,20]. Prolyl hydroxylated HIF-α is ubiquitinated by the ubiquitin E3 ligase complex VHL/Cullin-2/Elongin-B/C and then degraded at the 26*S* proteasome. In the absence of O_2_, HIF-α is no longer hydroxylated and ubiquitinated, thereby escaping from proteasomal degradation. Upon stabilization, HIF-α dimerizes with the HIF-1β subunit in the nucleus, and the heterodimeric HIF binds the hypoxia response element (5′-A/GCGTG-3′, HRE) to enhance gene transcription. Previous studies have shown that HIF induces the transcription of genes encoding glucose transporters and glycolytic enzymes in GBM, thereby providing a metabolic advantage for tumor cell growth [21]. Cancer cells also undergo reductive glutamine metabolism under hypoxia to generate glutamate and α-ketoglutarate, which are necessary to maintain the TCA cycle and to facilitate lipid synthesis [22]. Hypoxia-induced reductive glutamine metabolism is HIF-2-dependent [22]. We recently showed that HIF-1 induces BCAT1 to reprogram branched-chain amino acid metabolism and glutamate production in GBM cells [23]. However, the role of HIF in other aminotransferases remains unknown.

In this study, we report that GPT2 is directly induced by HIF-2 in human GBM. Genetic inhibition of GPT2 decreases GBM cell growth and migration in vitro and tumor growth in mice. These findings reveal GPT2 as a new hypoxia-induced aminotransferase and a possible therapeutic target for the treatment of human GBM.

## 2. Materials and Methods

### 2.1. Plasmid Constructs

Human full-length *GPT2* cDNA was amplified by PCR and cloned into p3 × FLAG-CMV-7 or pLV-HA-EGFP vector. DNA oligonucleotides containing wildtype (WT) or mutant *GPT2* HRE (Supplementary Table S1) were annealed and ligated into MluI/BglII-linearized pGL2-promoter vector (Promega, Madison, WI, USA). DNA oligonucleotides of the single-guide RNA (sgRNA) targeting human *GPT2* (Supplementary Table S1) were annealed and ligated into BsmBI-linearized lentiCRISPRv2 vector (Addgene, Watertown, MA, USA, #52961). DNA oligonucleotides of the short hairpin RNA (shRNA) targeting human *GPT2* (Supplementary Table S1) were annealed and ligated into EcoRI/AgeI-linearized pLKO.1 vector (Addgene, Watertown, MA, USA, #8453). Other constructs have been described previously [23,24,25]. All recombinant plasmids were verified by nucleotide sequence analysis. 

### 2.2. Cell Culture and Transfection

HEK293T (gift from Gregg L. Semenza at Johns Hopkins University, Baltimore, MD, USA), U251MG (gift from Sandeep Burma at UT Health San Antonio, San Antonio, TX, USA), and U87MG (gift from Gregg L. Semenza at Johns Hopkins University, Baltimore, MD, USA) cells were cultured in DMEM supplemented with 10% heat-inactivated fetal bovine serum (FBS) at 37 °C in a 5% CO_2_/95% air incubator. Primary GBM spheres were cultured as described previously [23]. The study was approved by the Institutional Review Board at UT Southwestern Medical Center with informed consent (STU 082018-004). Hypoxic cells were cultured in DMEM supplemented with FBS in the absence or presence of HIF-2α inhibitor (10 μM, Sigma, St. Louis, MO, USA, Cat.#: SML0883) and placed in a modular incubator chamber (Billups-Rothenberg, San Diego, CA, USA) flushed with a gas mixture of 1% O_2_, 5% CO_2_, and balanced N_2_. Cells were transfected using PolyJet (SignaGen, Frederick, MD, USA) or FuGENE6 (Promega, Madison, WI, USA) transfection reagent according to the manufacturer’s protocol. All cell lines are mycoplasma-free and have been authenticated by STR DNA profiling analysis.

### 2.3. Generation of HIF-α Knockout (KO), GPT2 Knockdown (KD)/KO, and GPT2 Rescue Cell Lines

Lentivirus encoding HA-GPT2, GPT2 shRNA, or GPT2 sgRNA was prepared by transient transfection of HEK293T cells with shRNA or sgRNA transducing vector and packaging vectors pMD2.G and psPAX2 using PolyJet (SignaGen, Frederick, MD, USA). Cells were transduced with lentivirus encoding GPT2 shRNA or sgRNA and treated with puromycin. GPT2 rescue cells were generated by infecting GPT2 KO cells with HA-GPT2 lentivirus. HIF-1α KO, HIF-2α KO, and HIF-1/2α double KO (DKO) cells were generated by transfection with HIF-1α and/or HIF-2α sgRNAs as described previously [23]. GPT2 KD, KO, and rescue cells were verified by immunoblot assay. 

### 2.4. Colony Formation Assay

Cells were seeded on a 48-well plate and exposed to 20% or 1% O_2_ for 7–12 days. Colonies were washed with PBS, fixed with 4% paraformaldehyde, and stained with 0.01% crystal violet.

### 2.5. Cell Migration Assay

Cells were resuspended in serum-free medium, seeded in a transwell insert with complete medium containing 10% FBS at the bottom chamber, and exposed to 20% or 1% O_2_ for 16 h. After removal of remaining cells in the transwell insert, cells that migrated to the lower side of the transwell insert were fixed with methanol and stained with 0.01% crystal violet. After washing, the crystal violet dye was dissolved in 10% acetic acid and measured by a plate reader at OD_570 nm_.

### 2.6. RNA Sequencing (RNA-Seq)

U251MG cells were exposed to 20% or 1% O_2_ for 24 h. Total RNA was isolated using the RNeasy mini kit and treated with DNase (Qiagen, Hilden, Germany). RNA quality was confirmed by an RNA integrity number score of 8.5 or higher via Agilent Tapestation 4200. DNA-free RNA was used for library preparation with a Kapa mRNA HyperPrep Kit (Roche, Wilmington, MA, USA) and sequenced on the Illumina NextSeq 500 with the read configuration as 76 bp, single end. Differential expression of genes was analyzed as described previously [24].

### 2.7. Quantitative Reverse Transcription-Polymerase Chain Reaction (qRT-PCR) Assay 

RT-qPCR assays were performed as described previously [26]. Briefly, total RNA was isolated from cultured cells using Trizol reagent (Thermo Fisher, Waltham, MA, USA), treated with DNase I (Ambion, Austin, TX, USA), and reverse transcribed using an iScript cDNA Synthesis Kit (Bio-Rad, Hercules, CA, USA). Real-time qPCR was performed with primers listed in Supplementary Table S2 in a CFX-96 Real-Time System (Bio-Rad) using iTaq Universal SYBR Green Supermix (Bio-Rad). Target mRNA expression was normalized to 18*S* rRNA and its fold change was calculated based on the threshold cycle (Ct) as 2^−Δ(ΔCt)^, where ΔCt = Ct_target_ − Ct_18S rRNA_ and Δ(ΔCt) = ΔCt_1% O2_ − ΔCt_20% O2_.

### 2.8. Immunoblot Assay

Cells or tissues were lysed in modified lysis buffer (50 mM Tris-HCl, pH 7.5, 150 mM NaCl, 1 mM β-mercaptoethanol, 1% Igepal, and protease inhibitor cocktail) for 30 min on ice, followed by centrifugation at 13,000 *g* for 15 min at 4 °C. Equal amounts of lysates were fractionated by SDS-PAGE and subjected to immunoblot assays with the following antibodies: GPT2 (Proteintech, Rosemont, IL, USA, Cat.#: 16757-1-AP), FLAG (Sigma, St. Louis, MO, USA, Cat#: F3165), HIF-1α (homemade or BD Biosciences, San Jose, CA, USA Cat.#: 610958), HIF-2α (homemade or Novus Biologicals, Littleton, CO, USA, Cat.#: NB100-122), α-Tubulin (Santa Cruz Biotechnology, Dallas, TX, USA, Cat.#: sc-8035), Tom20 (Proteintech, Rosemont, IL, USA, Cat.#: 11802-1-AP), histone H3 (Novus Biologicals, Littleton, CO, USA, Cat.#: NB500-267), H3K9me3 (Abcam, Waltham, MA, USA, Cat.#: ab8898), H3K9me2 (Cell Signaling Technology, Danvers, MA, USA, Cat.#: 4658S), H3K9me (Sigma, St. Louis, MO, USA, Cat.#: 07-450), H3K4me3 (Sigma, St. Louis, MO, USA, Cat.#: 07-473), H3K4me2 (Cell Signaling Technology, Danvers, MA, USA, Cat.#: 9725S), H3K4me (Cell Signaling Technology, Danvers, MA, USA, Cat.#: 5326S), ITGA6 (Cell Signaling Technology, Danvers, MA, USA, Cat.#: 3750S), or actin (Proteintech, Rosemont, IL, USA, Cat.#: 66009-1-Ig). 

### 2.9. Subcellular Fractionation Assay

U251MG cells were exposed to 20% or 1% O_2_ for 24 h and lysed in hypotonic buffer (10 mM HEPES/KOH, pH 7.5, 10 mM KCl, 1.5 mM MgCl_2_, 1 mM K_2_EDTA, 1 mM EGTA, 0.1% Igepal, 1 mM DTT, and protease inhibitor cocktail), incubated on ice for 30 min, and disrupted in a Dounce homogenizer. After removing intact cells by centrifugation at 50× *g* for 10 min at 4 °C, the supernatant was subjected to centrifugation at 800× *g* for 10 min at 4 °C. The resulting nuclear pellet was washed twice with isotonic buffer (hypotonic buffer plus 250 mM sucrose) and lysed in isotonic buffer by sonication. The supernatant was subjected to centrifugation at 6000× *g* for 20 min at 4 °C to separate mitochondria (pellet) and cytosol (supernatant). The mitochondrial pellet was washed twice with isotonic buffer and lysed in isotonic buffer by sonication. Equal amounts of lysates were used for immunoblotting assay.

### 2.10. α-Ketoglutarate Assay

The cellular α-ketoglutarate levels were measured using a α-ketoglutarate colorimetric/fluorometric assay kit (Cat.#: K677-100, Biovision, Milpitas, CA, USA), as described previously [27]. Cells were lysed in ice-cold a-ketoglutarate assay buffer, sonicated, and centrifuged at 13,000 rpm for 10 min at 4 °C. The supernatant was filtered using a 10 kDa spin column (Millipore, Burlington, MA, USA) and subjected to measurement at 535/587 nm in a Tecan Spark 10M plate reader according to the manufacturer’s instructions. The α-ketoglutarate levels were normalized to cellular protein concentrations quantified by Bradford assay (Bio-Rad, Hercules, CA, USA).

### 2.11. Luciferase Reporter Assay

Luciferase reporter assays were performed as described previously [26]. Briefly, HEK293T cells were plated on poly-L-lysine-coated 48-well plates and transiently transfected with WT or mutant *GPT2* HRE reporter plasmid or pGL2-promoter empty vector (EV) and control reporter plasmid pSV40-Renilla. After 24 h, cells were exposed to 20% or 1% O_2_ for 24 h. The firefly and *Renilla* luciferase activities were measured using the Dual Luciferase Reporter Assay System (Promega, Madison, WI, USA) according to the manufacturer’s protocol. 

### 2.12. ChIP-qPCR Assay

ChIP-qPCR assays were performed as described previously [26]. U251MG cells were exposed to 20% or 1% O_2_ for 24 h, crosslinked with 1% formaldehyde for 20 min at room temperature, and quenched in 0.125 M glycine. Cells were lysed in lysis buffer (50 mM Tris-HCl, 10 mM EDTA, 1% SDS, protease inhibitor cocktail), sonicated, and subjected to immunoprecipitation in the presence of salmon sperm DNA/protein A beads with antibodies against HIF-1α, HIF-2α, HIF-1β, or IgG overnight at 4 °C. Precipitated chromatin DNA was extensively washed, eluted with freshly prepared elution buffer (0.1 M NaHCO_3_, 1% SDS), decrosslinked at 65 °C for 4 h, incubated with proteinase K at 45 °C for 45 min, purified using phenol/chloroform/isoamyl alcohol (25:24:1, *v*/*v*), and quantified by real-time qPCR. The primers used for ChIP-qPCR are listed in Supplementary Table S2. Fold enrichment was calculated based on Ct as 2^−Δ(ΔCt)^, where ΔCt = Ct_IP_ − Ct_Input_ and Δ(ΔCt) = ΔCt_antibody_ − ΔCt_IgG_. 

### 2.13. Animal Studies

Animal studies were approved by the Animal Care and Use Committee at UT Southwestern Medical Center (APN2017-102199). Scrambled control (SC) and GPT2 KD#2 U87MG cells (3 million) were mixed with Matrigel (1:1) and subcutaneously implanted into the flank side of 6- to 8-week-old male NOD/SCID mice (Envigo, Indianapolis, IN, USA). Tumor volume was measured with a caliper as described previously [27]. 

### 2.14. Statistical Analysis

Data were repeated for at least three times and expressed as mean ± SEM. Statistical analysis was performed by Student’s *t* test between two groups, and one-way or two-way ANOVA with multiple testing correction within multiple groups. *p* < 0.05 is considered significant.

## 3. Results

### 3.1. GPT2 Is Induced by Hypoxia in Human GBM Cell Lines and Tumors

**Figure 1:**
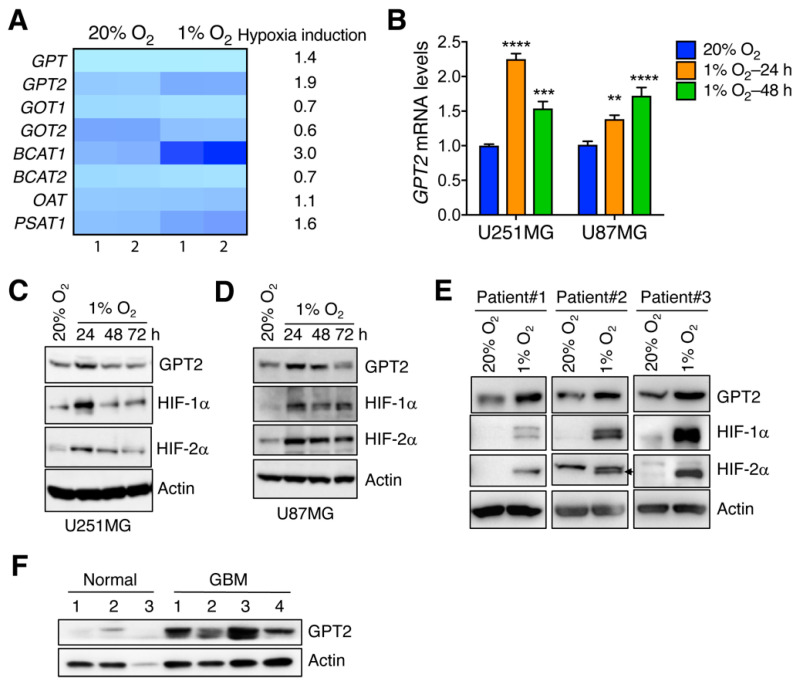
Hypoxia induces GPT2 expression in GBM cells. (**A**) RNA-seq analysis of eight aminotransferases in U251MG cells exposed to 20% or 1% O_2_ for 24 h. The color indicates normalized mRNA expression levels. Fold changes in mRNAs by hypoxia are shown on the right. The sample numbers are shown at the bottom. (**B**) qRT-PCR analysis of *GPT2* mRNA in U251MG and U87MG cells exposed to 20% or 1% O_2_ for 24 or 48 h (mean ± SEM, *n* = 3–6). ** *p* < 0.01, *** *p* < 0.001, **** *p* < 0.0001 vs. 20% O_2_ by one-way ANOVA with Dunnett’s test. (**C**) Immunoblot analysis of indicated proteins in U251MG cells exposed to 20% or 1% O_2_ for 24–72 h. (**D**) Immunoblot analysis of indicated proteins in U87MG cells exposed to 20% or 1% O_2_ for 24–72 h. (**E**) Immunoblot analysis of indicated proteins in patient-derived GBM spheres exposed to 20% or 1% O_2_ for 48 h. (**F**) Immunoblot analysis of indicated proteins in normal human brain and human GBM tissues. The sample numbers are shown on the top.

To study whether hypoxia induces glutamate-linked aminotransferases, we performed RNA-seq in human GBM U251MG cells exposed to 20% or 1% O_2_ for 24 h, and found that among eight aminotransferases, BCAT1 and GPT2 were the top two enzymes induced by hypoxia in U251MG cells (Figure 1A). We recently characterized hypoxia-induced BCAT1 in GBM cells [23], and thus, GPT2 is exclusively focused on here for further study. To validate these findings, we performed RT-qPCR in U251MG cells exposed to 20% or 1% O_2_ for 24 and 48 h. *GPT2* mRNA levels were significantly upregulated by hypoxia in a time-dependent manner (Figure 1B). Similar results were also observed in another human GBM U87MG cell line (Figure 1B). In line with mRNA upregulation, GPT2 protein levels were elevated after 24 h of hypoxia and then gradually decreased in both U251MG and U87MG cells, similar to HIF-1α and HIF-2α proteins (Figure 1C,D). We detected increased GPT2 protein levels in primary human GBM cells after exposure to 1% O_2_ for 48 h (Figure 1E). Consistently, GPT2 protein levels were significantly increased in human GBM tissues as compared with normal brain tissues (Figure 1F), indicating clinical relevance of GPT2 induction in human GBM. Together, these data indicate that GPT2 is induced by hypoxia in human GBM.

### 3.2. HIF-2 Is Required for Hypoxia-Induced GPT2 Expression in GBM Cells 

**Figure 2:**
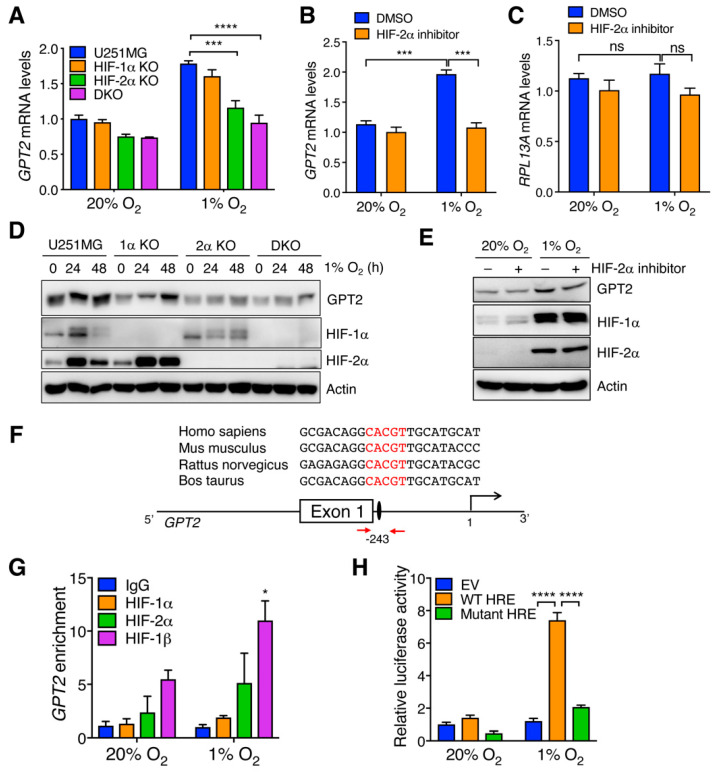
*GPT2* is a direct HIF-2 target gene. (**A**) qRT-PCR analysis of *GPT2* mRNA in parental, HIF-1α KO, HIF-2α KO, and HIF-1/2α DKO U251MG cells exposed to 20% or 1% O_2_ for 24 h (mean ± SEM, *n* = 3). *** *p* < 0.001, **** *p* < 0.0001 by two-way ANOVA with Tukey’s test. (**B**,**C**) qRT-PCR analysis of *GPT2* (**B**) and *RPL13A* (**C**) mRNA in U87MG cells exposed to 20% or 1% O_2_ for 24 h in the absence or presence of HIF-2α inhibitor (mean ± SEM, *n* = 3). *** *p* < 0.001 by two-way ANOVA with Tukey’s test. ns, not significant. (**D**) Immunoblot analysis of indicated proteins in parental, HIF-1α KO, HIF-2α KO, and HIF-1/2α DKO U251MG cells exposed to 20% or 1% O_2_ for 24 or 48 h. (**E**) Immunoblot analysis of indicated proteins in U87MG cells exposed to 20% or 1% O_2_ for 24 h in the absence or presence of HIF-2α inhibitor. (**F**) The scheme of the human *GPT2* HRE. The nucleotide sequences of the HRE (shown in red) from human, mouse, rat, and bovine are shown on the top. The transcription start site is designated as 1. Exon and intron are not drawn to scale. Red arrows indicate the locations of qPCR primers. (**G**) ChIP-qPCR analysis of enrichment of HIF-1α, HIF-2α, and HIF-1β, and control IgG at the *GPT2* HRE in U251MG cells exposed to 20% or 1% O_2_ for 24 h (mean ± SEM, *n* = 3). * *p* < 0.05 vs. 20% O_2_ by two-way ANOVA with Sidak’s test. (**H**) HEK293T cells were cotransfected with empty pGL2p vector (EV), pGL2p-GPT2 WT HRE, or pGL2p-GPT2 mutant HRE, and pSV40-Renilla, and exposed to 20% or 1% O_2_ for 24 h. The ratio of firefly/Renilla activities was normalized to EV at 20% O_2_ (mean ± SEM, *n* = 3). **** *p* < 0.0001 by two-way ANOVA with Tukey’s test.

To determine whether HIF is required for hypoxia-induced GPT2 expression in GBM cells, we performed qRT-PCR in parental, HIF-1α KO, HIF-2α KO, HIF-1α, and HIF-2α DKO U251MG cells exposed to 20% or 1% O_2_ for 24 h. As expected, *GPT2* mRNA was induced by hypoxia in parental U251MG cells, which was eliminated by HIF-2α KO and HIF-1/2α DKO, but not HIF-1α KO (Figure 2A). Genetic inhibition of HIF-2 by a specific HIF-2α inhibitor (10 μM) also abolished hypoxia-induced *GPT2* mRNA expression in U87MG cells (Figure 2B). However, the non-HIF target gene *RPL13A* was not affected by HIF-2α inhibitor treatment in U87MG cells (Figure 2C), confirming the specificity of our assay. Consistently, HIF-2α KO and HIF-1/2α DKO abolished hypoxia-induced GPT2 protein expression in U251MG cells, whereas HIF-1α KO failed to do so (Figure 2D). Likewise, treatment of HIF-2α inhibitor (10 μM) decreased GPT2 levels in hypoxic U87MG cells (Figure 2E). These data indicate that hypoxia upregulates GPT2 expression in GBM cells in a HIF-2-dependent manner.

To determine whether *GPT2* is a direct HIF-2 target gene, we analyzed the DNA sequence at the 5′ untranslated region of human, mouse, rat, and bovine *GPT2* gene, and found a conserved HRE (5′-ACGTG-3′ on the antisense strand) in all these species (Figure 2F). To determine whether HIF-2 directly binds to this HRE, we performed ChIP-qPCR in U251MG cells exposed to 20% or 1% O_2_ for 24 h, and found that HIF-2α and HIF-1β were enriched at the *GPT2* HRE and their enrichment was increased by hypoxia (Figure 2G). Much stronger binding of HIF-1β to the HRE is possibly due to more HIF-1β protein precipitation by its antibody we used. In contrast, HIF-1α did not occupy this HRE in U251MG cells (Figure 2G). To examine whether this HRE is functional, a 55-base pair DNA fragment containing the HRE was cloned into the upstream of the SV40 promoter and firefly luciferase gene in a pGL2p reporter vector. HEK293T cells were transfected with pGL2p EV or pGL2p-GPT2 HRE and the control plasmid pSV40-Renilla, and exposed to 20% or 1% O_2_ for 24 h. HEK293T cells was used in this assay because of their high transfection efficiency. *GPT2* HRE significantly stimulated the expression of firefly luciferase gene but not control *Renilla* gene in HEK293T cells under hypoxia as compared with the EV (Figure 2H). Mutation of 5′-CGT-3′ into 5′-AAA-3′ within the *GPT2* HRE completely abolished hypoxia-induced firefly luciferase activity in HEK293T cells (Figure 2H). Together, these data indicate that *GPT2* is a direct HIF-2 target gene.

### 3.3. Altered α-Ketoglutarate by GPT2 Fails to Regulate HIF-α Protein Stability and Histone Lysine Methylation in GBM Cells

**Figure 3:**
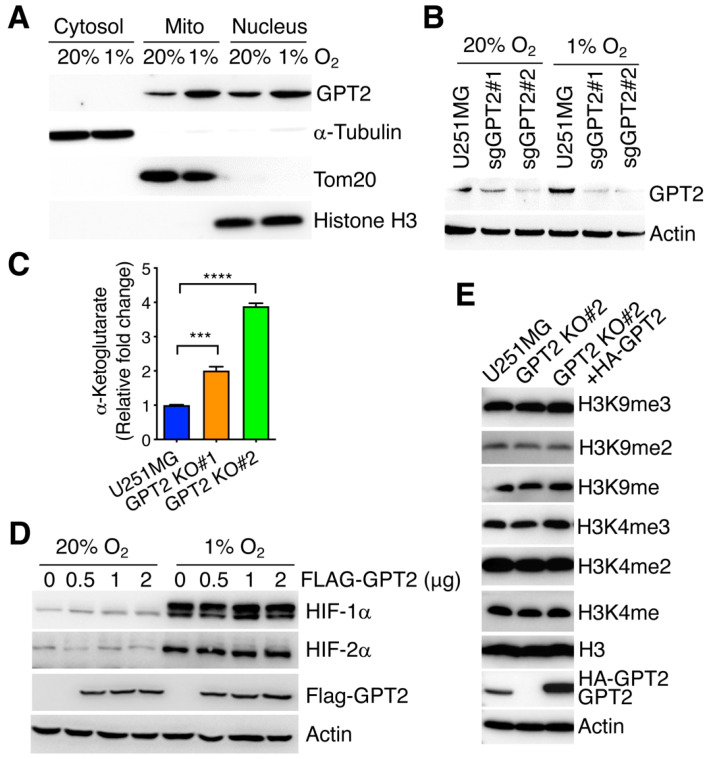
GPT2 has no effect on levels of HIF and histone lysine methylation. (**A**) Immunoblot analysis of indicated proteins in cytosolic, mitochondrial (mito), and nuclear fractionations isolated from U251MG cells exposed to 20% or 1% O_2_ for 24 h. (**B**) Immunoblot analysis of indicated proteins in parental and GPT2 KO U251MG cells exposed to 20% or 1% O_2_ for 24 h. (**C**) Quantification of intracellular α-ketoglutarate levels in parental and GPT2 KO#1 or #2 U251MG cells (mean ± SEM, *n* = 3). *** *p* < 0.001, **** *p* < 0.0001 by one-way ANOVA with Dunnett’s test. (**D**) Immunoblot analysis of indicated proteins in U251MG cells transfected with the different amount of FLAG-GPT2 plasmid and exposed to 20% or 1% O_2_ for 6 h. (**E**) Immunoblot analysis of indicated proteins in parental, GPT2 KO#2, and GPT2 rescue U251MG cells.

Next, we studied the cellular localization of GPT2 by extracting cytosolic, mitochondrial, and nuclear fractionations from U251MG cells exposed to 20% or 1% O_2_ for 24 h. The purity of these three fractionations was confirmed by analyzing the expression of their respective markers α-tubulin, Tom20, and histone H3 (Figure 3A). GPT2 localized in the nucleus and mitochondria but not cytosol (Figure 3A). Both mitochondrial and nuclear GPT2 proteins were induced by hypoxia in U251MG cells (Figure 3A). To determine whether GPT2 regulates intracellular α-ketoglutarate levels in GBM cells, we generated GPT2 KO U251MG cells by the CRISPR/Cas9 technique (Figure 3B). GPT2 KO by either of the two sgRNAs significantly increased intracellular α-ketoglutarate levels in U251MG cells (Figure 3C). α-ketoglutarate is an important cofactor for dioxygenases including PHDs and histone demethylases Jumonji domain-containing proteins [28]. To determine whether GPT2 regulates the activity of α-ketoglutarate-dependent dioxygenases, we assessed the protein levels of HIF-1α and HIF-2α, two substrates of PHDs. Ectopic expression of GTP2 failed to alter the protein levels of HIF-1α and HIF-2α in U251MG cells under 20% and 1% O_2_ (Figure 3D). Likewise, mono-, di-, and tri-methyl lysine (K) 4 and 9 of histone H3 were also not affected by KO or expression of GPT2 in U251MG cells (Figure 3E). These results suggest that altered α-ketoglutarate by GPT2 is unlikely to affect the activity of PHDs and H3K4 or H3K9 demethylases in GBM cells.

### 3.4. GPT2 Increases GBM Cell Migration under Hypoxia

**Figure 4:**
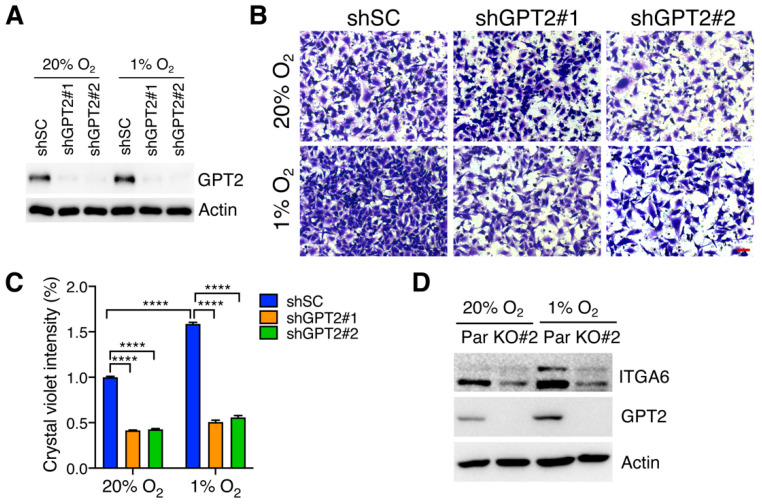
GPT2 promotes GBM cell migration in vitro. (**A**) Immunoblot analysis of indicated proteins in SC and GPT2 knockdown U251MG cells exposed to 20% or 1% O_2_ for 24 h. (**B**,**C**) Migration of SC and GPT2 knockdown U251MG cells under 20% and 1% O_2_. Representative images are shown in (**B**). Scale bar, 50 μm. Cell migration was quantified in (**C**) (mean ± SEM, *n* = 3). **** *p* < 0.0001 by two-way ANOVA with Tukey’s test. (**D**) Immunoblot analysis of indicated proteins in parental (Par) and GPT2 KO#2 U251MG cells exposed to 20% or 1% O_2_ for 48 h.

We next studied the effect of GPT2 on GBM cell migration by the Boyden transwell assay. We generated two independent GPT2 KD U251MG cell lines by shRNAs (Figure 4A). GPT2 KD by either of its shRNAs significantly decreased migration of U251MG cells under 20% and 1% O_2_ as compared with shSC (Figure 4B,C). Similar results were also observed in U251MG cells transduced with sgGPT2#1 or #2 (Supplementary Figure S1). Consistently, GPT2 KO decreased the expression of ITGA6, a HIF-induced integrin family member involved in cancer cell motility [29], in U251MG cells under 20% and 1% O_2_ (Figure 4D). These data indicate that GPT2 promotes migration of U251MG cells in vitro.

### 3.5. GPT2 Promotes GBM Growth In Vitro and in Mice

**Figure 5:**
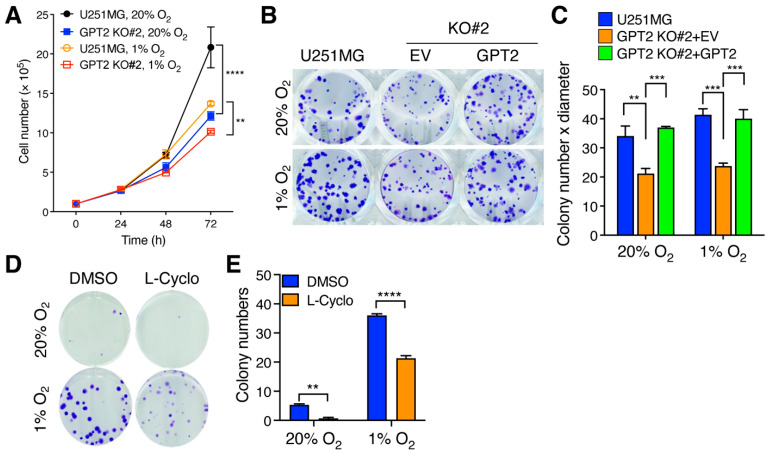
GPT2 promotes GBM cell growth in vitro. (**A**) Proliferation of parental and GPT2 KO U251MG cells exposed to 20% and 1% O_2_ (mean ± SEM, *n* = 3). ** *p* < 0.01, **** *p* < 0.0001 by two-way ANOVA with Tukey’s test. (**B**,**C**) Colony formation of parental, GPT2 KO, and GPT2 rescue U251MG cells under 20% and 1% O_2_. Representative images are shown in (**B**). Quantification of colony growth is shown in (**C**) (mean ± SEM, *n* = 3). ** *p* < 0.01, *** *p* < 0.001 by two-way ANOVA with Tukey’s test. (**D**,**E**) Colony formation of U251MG cells exposed to 20% or 1% O_2_ in the presence of DMSO or GPT2 inhibitor L-Cyclo (50 μM). Representative images are shown in (**D**). Quantification of colony number is shown in (**E**) (mean ± SEM, *n* = 3). ** *p* < 0.01, **** *p* < 0.0001 by two-way ANOVA with Tukey’s test.

Next, we studied the effect of GPT2 on cell proliferation in vitro. While chronic hypoxia (>24 h) inhibited proliferation of U251MG cells under 2D culture conditions, GPT2 KO significantly decreased U251MG cell growth in a time-dependent manner under 20% and 1% O_2_ (Figure 5A). We further employed colony formation assay to validate the role of GPT2 in GBM cell growth. Hypoxia increased colony formation of U251MG cells and GPT2 KO decreased U251MG colony formation under 20% and 1% O_2_ (Figure 5B,C). Similar results were also found in U87MG cells (Supplementary Figure S2). Re-expression of HA-GPT2 in GPT2 KO#2 U251MG cells rescued colony formation under 20% and 1% O_2_ (Figure 5B,C). In line with genetic studies, treatment of a GPT2 pharmacological inhibitor L-Cycloserine (L-Cyclo, 50 μM) robustly decreased colony formation of U251MG cells under 20% and 1% O_2_ (Figure 5D,E). These data indicate that GPT2 promotes GBM cell growth in vitro.

**Figure 6:**
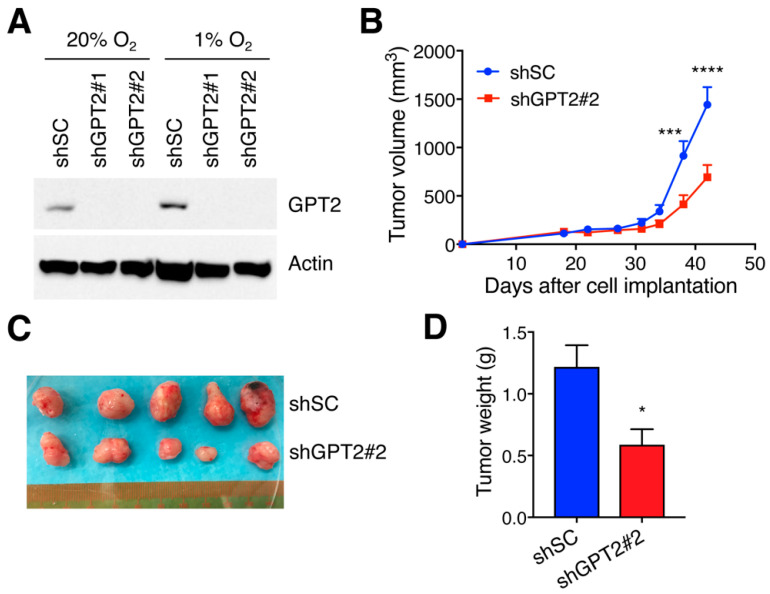
GPT2 promotes GBM tumor growth in mice. (**A**) Immunoblot analysis of indicated proteins in SC and GPT2 knockdown U87MG cells exposed to 20% or 1% O_2_ for 24 h. (**B**–**D**) Growth of SC and GPT2 knockdown U87MG tumors in mice. The tumor growth curve is shown in (**B**) (mean ± SEM, *n* = 5). *** *p* < 0.001, **** *p* < 0.0001 by two-way ANOVA with Sidak’s test. The tumor image is shown in (**C**). The tumor weight is shown in (**D**) (mean ± SEM, *n* = 5). * *p* < 0.05 by Student’s *t* test.

We next generated GPT2 KD U87MG cells (Figure 6A) and subcutaneously implanted shSC or GPT2 KD#2 U87MG cells into NOD/SCID mice. GPT2 KD in U87MG cells significantly decreased primary tumor growth in mice as compared with SC (Figure 6B–D). Our U251MG cells were not able to grow in NOD/SCID mice, which prevented us from validating U87MG tumor data. Nevertheless, these findings indicate that GPT2 promotes tumor growth in mice.

## 4. Discussion

In the present study, we demonstrated the oncogenic role of GPT2 in human GBM. We found GPT2 upregulation in human GBM, which was possibly controlled by hypoxia, a hallmark of GBM tumor microenvironment. We further identified *GPT2* as a direct HIF-2 target gene in human GBM through multiple biochemical approaches. Inconsistent GPT2 mRNA and protein expression patterns at 48 h after hypoxia in U87MG cells suggest that hypoxia may control GPT2 mRNA translation as well. Interestingly, HIF-1 has little effect on GPT2 expression in GBM. Both HIF-1 and HIF-2 are strongly activated in cancer cells in response to hypoxia and share many conserved roles in cancer progression [14]. However, HIF-1 and HIF-2 also have their unique functions in cancer cells [22,23]. A previous study identified a specific role of HIF-2 in reductive glutamine metabolism [22]. Our recent work revealed the predominant regulation of BCAT1 expression by HIF-1 in GBM cells [23]. Our ChIP-qPCR assay shows that HIF-2α but not HIF-1α is enriched at the promoter of *GPT2* in GBM cells under hypoxia, suggesting that the epigenetic mechanism may be responsible for HIF-2α recruitment to the *GPT2* gene and subsequent GPT2 expression.

A previous report showed that GPT2 is induced by ATF4 under endoplasmic reticulum stress [10]. Activation of ATF4 links metabolic changes and cell survival in GBM [30]. ATF4 protein is stabilized and activated by hypoxia [31]. Although our results revealed a direct role of HIF-2 in GPT2 induction in hypoxic GBM cells, we cannot exclude a possible indirect regulation of GPT2 by ATF4 in our study.

GPT2 is known to be a mitochondrial protein. We show here that GPT2 is localized in the nucleus, in addition to mitochondria. The function of nuclear GPT2 remains unknown. Although GPT2 regulates the intracellular α-ketoglutarate levels, it fails to alter the levels of HIF-1α, HIF-2α, and methylation of H3K4 and H3K9 in GBM cells. Further studies are needed to characterize whether nuclear GPT2 regulates the global and local changes in histone lysine methylation and DNA methylation across the genome via α-ketoglutarate-dependent dioxygenases. Interestingly, we observed reduced ITGA6 levels in GPT2 KO U251MG cells, consistent with impaired cell migration. These findings implicate a critical role of nuclear GPT2 in gene regulation and GBM pathogenesis.

Glutamate is an important neurotransmitter regulating synaptic plasticity in brain and also an energy source for GBM cell proliferation. GPT2 is one of the important enzymes controlling cellular glutamate and glutamine levels, which are elevated in human GBM [32]. Previous studies in colorectal cancer showed that PIK3CA mutations reprogram glutamine metabolism by upregulating GPT2 in colorectal cancer cells, making these cancer cells more dependent on glutamine for their survival [33]. Nuclear receptor liver receptor homolog 1, a key regulator in the process of hepatic tumorigenesis, also relies on GPT2 for its role in glutamine metabolism [34]. HIF is a critical regulator of metabolism of glutamine and glutamate in cancer cells [22,23,32]. Here, we showed that GPT2 promotes GBM cell migration and growth in vitro and in vivo. Whether or not GPT2 regulates HIF-mediated alterations in glutamate and glutamine metabolism, leading to GBM progression, requires future investigation.

In conclusion, our work revealed that GPT2 is an oncogenic metabolic enzyme and controlled by HIF in human GBM cells. Targeting GPT2 may have a therapeutic benefit in human GBM.

## Data Availability

All data supporting the conclusions in the paper are available upon reasonable request.

## Supplementary Materials

The following supporting information can be downloaded at: https://www.mdpi.com/article/10.3390/cells11162597/s1, Table S1: The sequences of sgRNAs, shRNAs, and HRE; Table S2: The sequences of qPCR primers; Figure S1: GPT2 promotes GBM cell migration in vitro; Figure S2: GPT2 promotes GBM cell growth in vitro.

## References

[1] Deshmukh R., Allega M.F., Tardito S. (2021). A map of the altered glioma metabolism. Trends Mol. Med..

[2] Jung E., Alfonso J., Osswald M., Monyer H., Wick W., Winkler F. (2019). Emerging intersections between neuroscience and glioma biology. Nat. Neurosci..

[3] Maus A., Peters G.J. (2017). Glutamate and alpha-ketoglutarate: Key players in glioma metabolism. Amino Acids.

[4] DeBerardinis R.J., Mancuso A., Daikhin E., Nissim I., Yudkoff M., Wehrli S., Thompson C.B. (2007). Beyond aerobic glycolysis: Transformed cells can engage in glutamine metabolism that exceeds the requirement for protein and nucleotide synthesis. Proc. Natl. Acad. Sci. USA.

[5] Takano T., Lin J.H., Arcuino G., Gao Q., Yang J., Nedergaard M. (2001). Glutamate release promotes growth of malignant gliomas. Nat. Med..

[6] Kim M., Gwak J., Hwang S., Yang S., Jeong S.M. (2019). Mitochondrial GPT2 plays a pivotal role in metabolic adaptation to the perturbation of mitochondrial glutamine metabolism. Oncogene.

[7] Yang R.Z., Blaileanu G., Hansen B.C., Shuldiner A.R., Gong D.W. (2002). cDNA cloning, genomic structure, chromosomal mapping, and functional expression of a novel human alanine aminotransferase. Genomics.

[8] Celis K., Shuldiner S., Haverfield E.V., Cappell J., Yang R., Gong D.W., Chung W.K. (2015). Loss of function mutation in glutamic pyruvate transaminase 2 (GPT2) causes developmental encephalopathy. J. Inherit. Metab. Dis..

[9] Ouyang Q., Nakayama T., Baytas O., Davidson S.M., Yang C., Schmidt M., Lizarraga S.B., Mishra S., Ei-Quessny M., Niaz S. (2016). Mutations in mitochondrial enzyme GPT2 cause metabolic dysfunction and neurological disease with developmental and progressive features. Proc. Natl. Acad. Sci. USA.

[10] Salgado M.C., Meton I., Anemaet I.G., Baanante I.V. (2014). Activating transcription factor 4 mediates up-regulation of alanine aminotransferase 2 gene expression under metabolic stress. Biochim. Biophys. Acta.

[11] Itkonen H.M., Gorad S.S., Duveau D.Y., Martin S.E., Barkovskaya A., Bathen T.F., Moestue S.A., Mills I.G. (2016). Inhibition of O-GlcNAc transferase activity reprograms prostate cancer cell metabolism. Oncotarget.

[12] Coss C.C., Bauler M., Narayanan R., Miller D.D., Dalton J.T. (2012). Alanine aminotransferase regulation by androgens in non-hepatic tissues. Pharm. Res..

[13] Luo W., Wang Y. (2019). Hypoxia Mediates Tumor Malignancy and Therapy Resistance. Adv. Exp. Med. Biol..

[14] Semenza G.L. (2012). Hypoxia-inducible factors: Mediators of cancer progression and targets for cancer therapy. Trends Pharmacol. Sci..

[15] Wang G.L., Jiang B.H., Rue E.A., Semenza G.L. (1995). Hypoxia-inducible factor 1 is a basic-helix-loop-helix-PAS heterodimer regulated by cellular O_2_ tension. Proc. Natl. Acad. Sci. USA.

[16] Tian H., McKnight S.L., Russell D.W. (1997). Endothelial PAS domain protein 1 (EPAS1), a transcription factor selectively expressed in endothelial cells. Genes Dev..

[17] Gu Y.Z., Moran S.M., Hogenesch J.B., Wartman L., Bradfield C.A. (1998). Molecular characterization and chromosomal localization of a third alpha-class hypoxia inducible factor subunit, HIF3alpha. Gene Expr..

[18] Ivan M., Kondo K., Yang H., Kim W., Valiando J., Ohh M., Salic A., Asara J.M., Lane W.S., Kaelin W.G. (2001). HIFalpha targeted for VHL-mediated destruction by proline hydroxylation: Implications for O_2_ sensing. Science.

[19] Epstein A.C., Gleadle J.M., McNeill L.A., Hewitson K.S., O’Rourke J., Mole D.R., Mukherji M., Metzen E., Wilson M.I., Dhanda A. (2001). *C. elegans* EGL-9 and mammalian homologs define a family of dioxygenases that regulate HIF by prolyl hydroxylation. Cell.

[20] Maxwell P.H., Wiesener M.S., Chang G.W., Clifford S.C., Vaux E.C., Cockman M.E., Wykoff C.C., Pugh C.W., Maher E.R., Ratcliffe P.J. (1999). The tumour suppressor protein VHL targets hypoxia-inducible factors for oxygen-dependent proteolysis. Nature.

[21] Gabriely G., Wheeler M.A., Takenaka M.C., Quintana F.J. (2017). Role of AHR and HIF-1alpha in Glioblastoma Metabolism. Trends Endocrinol. Metab..

[22] Metallo C.M., Gameiro P.A., Bell E.L., Mattaini K.R., Yang J., Hiller K., Jewell C.M., Johnson Z.R., Irvine D.J., Guarente L. (2011). Reductive glutamine metabolism by IDH1 mediates lipogenesis under hypoxia. Nature.

[23] Zhang B., Chen Y., Shi X., Zhou M., Bao L., Hatanpaa K.J., Patel T., DeBerardinis R.J., Wang Y., Luo W. (2021). Regulation of branched-chain amino acid metabolism by hypoxia-inducible factor in glioblastoma. Cell Mol. Life Sci..

[24] Chen Y., Zhang B., Bao L., Jin L., Yang M., Peng Y., Kumar A., Wang J.E., Wang C., Zou X. (2018). ZMYND8 acetylation mediates HIF-dependent breast cancer progression and metastasis. J. Clin. Investig..

[25] Bao L., Chen Y., Lai H.T., Wu S.Y., Wang J.E., Hatanpaa K.J., Raisanen J.M., Fontenot M., Lega B., Chiang C.M. (2018). Methylation of hypoxia-inducible factor (HIF)-1alpha by G9a/GLP inhibits HIF-1 transcriptional activity and cell migration. Nucleic Acids Res..

[26] Luo W., Hu H., Chang R., Zhong J., Knabel M., O’Meally R., Cole R.N., Pandey A., Semenza G.L. (2011). Pyruvate kinase M2 is a PHD3-stimulated coactivator for hypoxia-inducible factor 1. Cell.

[27] Zhang B., Peng H., Zhou M., Bao L., Wang C., Cai F., Zhang H., Wang J.E., Niu Y., Chen Y. (2022). Targeting BCAT1 combined with alpha-ketoglutarate triggers metabolic synthetic lethality in glioblastoma. Cancer Res..

[28] Losman J.A., Koivunen P., Kaelin W.G. (2020). 2-Oxoglutarate-dependent dioxygenases in cancer. Nat. Rev. Cancer.

[29] Brooks D.L., Schwab L.P., Krutilina R., Parke D.N., Sethuraman A., Hoogewijs D., Schorg A., Gotwald L., Fan M., Wenger R.H. (2016). ITGA6 is directly regulated by hypoxia-inducible factors and enriches for cancer stem cell activity and invasion in metastatic breast cancer models. Mol. Cancer.

[30] Ishida C.T., Zhang Y., Bianchetti E., Shu C., Nguyen T.T.T., Kleiner G., Sanchez-Quintero M.J., Quinzii C.M., Westhoff M.A., Karpel-Massler G. (2018). Metabolic Reprogramming by Dual AKT/ERK Inhibition through Imipridones Elicits Unique Vulnerabilities in Glioblastoma. Clin. Cancer Res..

[31] Koditz J., Nesper J., Wottawa M., Stiehl D.P., Camenisch G., Franke C., Myllyharju J., Wenger R.H., Katschinski D.M. (2007). Oxygen-dependent ATF-4 stability is mediated by the PHD3 oxygen sensor. Blood.

[32] Tanaka K., Sasayama T., Irino Y., Takata K., Nagashima H., Satoh N., Kyotani K., Mizowaki T., Imahori T., Ejima Y. (2015). Compensatory glutamine metabolism promotes glioblastoma resistance to mTOR inhibitor treatment. J. Clin. Investig..

[33] Hao Y.J., Samuels Y., Li Q.L., Krokowski D., Guan B.J., Wang C., Jin Z.C., Dong B.H., Cao B., Feng X.J. (2016). Oncogenic PIK3CA mutations reprogram glutamine metabolism in colorectal cancer. Nat. Commun..

[34] Xu P., Oosterveer M.H., Stein S., Demagny H., Ryu D., Moullan N., Wang X., Can E., Zamboni N., Comment A. (2016). LRH-1-dependent programming of mitochondrial glutamine processing drives liver cancer. Genes Dev..

