# Supplementary material for: GPT2 Is Induced by Hypoxia-Inducible Factor (HIF)-2 and Promotes Glioblastoma Growth
Source: Cells. 2022 Aug 20;11(16):2597. doi: 10.3390/cells11162597 (PMC9406858; doi:10.3390/cells11162597)
Supplement: Supplementary file 1 [file cells-11-02597-s001.zip › cells-1833735-supplementary.pdf]

cells

MDPI

Supplementary Material

GPT2 Is Induced by Hypoxia-Inducible Factor (HIF)-2 and Promotes Glioblastoma Growth

Bo Zhang [1,†], Yan Chen [1,†], Lei Bao [1] and Weibo Luo [1,2,*]

[1] Department of Pathology, UT Southwestern Medical Center, Dallas, TX 75390, USA
[2] Department of Pharmacology, UT Southwestern Medical Center, Dallas, TX 75390, USA
* Correspondence: weibo.luo@utsouthwestern.edu; Tel.: +1-214-645-4770
† Both authors contributed equally to this work.

Table S1. The sequences of sgRNAs, shRNAs, and HRE.

GPT2-sgRNA1	5′-GTCGTGAACCCCCCGGTGGC-3′
GPT2-sgRNA2	5′-AAGATCCTCGTCTCCGGGGG-3′
GPT2-shRNA1	5′-CGGCATTTCTACGATCCTGAA-3′
GPT2-shRNA2	5′-GACGCCATCCAGGTGAATTAC-3′
WT GPT2 HRE	5′-GTGCGGCGAGGGCCTACCAGGGGCGACAGGCACGTT GCATGCATGCCTTGGGCGC-3′
Mutant GPT2 HRE	5′-GTGCGGCGAGGGCCTACCAGGGGCGACAGGCTTTTTG CATGCATGCCTTGGGCGC-3′

Table S2. The sequences of qPCR primers.

GPT2 mRNA	Forward: 5′-ATGGCACTATGCACCTACCC-3′
	Reverse: 5′-CGGGCACGTTTCTTAGCATC-3′
RPL13A mRNA	Forward: 5′-CTCAAGGTCGTGCGTCTG-3′
	Reverse: 5′-TGGCTTTCTCTTTCCTCTTCTC-3′
18*S* rRNA	Forward:5′-CGGCGACGACCCATTCGAAC-3′
	Reverse: 5′-GAATCGAACCCTGATTCCCCGTC-3′
GPT2 HRE	Forward: 5′-CGCGAGCTAACCGAGTG-3′
	Reverse: 5′-ACACCCAACCGGCTTTC-3′

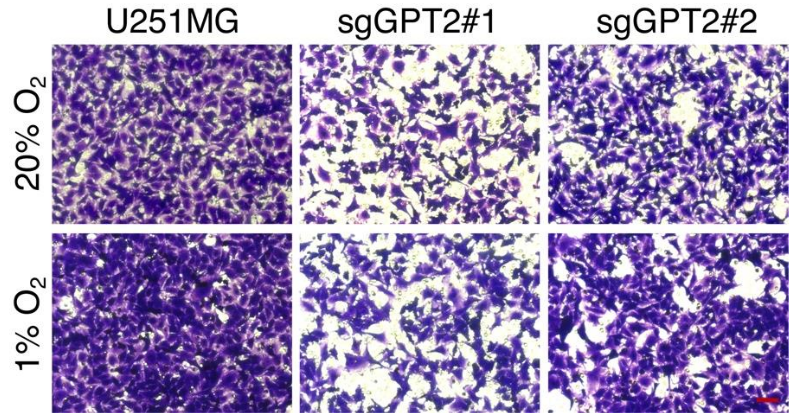

Figure S1. GPT2 promotes GBM cell migration in vitro. Parental and GPT2 knockout U251MG cells were seeded onto transwell inserts and exposed to 20% or 1% O_2 for 16 h. Representative cell migration images are shown. Scale bar, 50 μm.

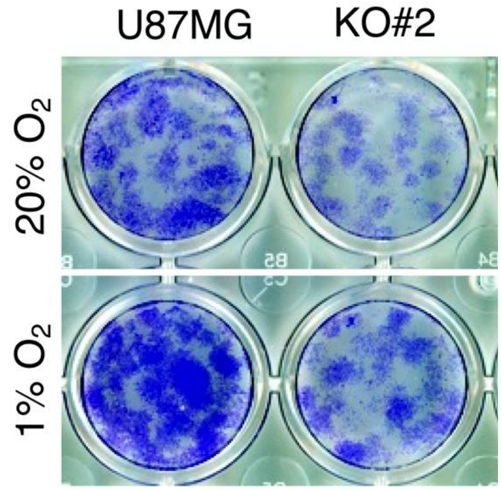

Figure S2. GPT2 promotes GBM cell growth in vitro. Parental and GPT2 KO#2 U87MG cells were exposed to 20% or 1% O_2 for 12 days. Colonies were stained with 0.01% crystal violet and imaged. Representative images are shown.
